# Pulmonary pathophysiology development of COVID-19 assessed by serial Electrical Impedance Tomography in the MaastrICCht cohort

**DOI:** 10.1038/s41598-022-18843-z

**Published:** 2022-08-25

**Authors:** Serge J. H. Heines, Bas C. T. van Bussel, Melanie J. Acampo-de Jong, Frank C. Bennis, Rob J. J. van Gassel, Rald V. M. Groven, Nanon F. L. Heijnen, Ben J. M. Hermans, René Hounjet, Johan van Koll, Mark M. G. Mulder, Marcel C. G. van de Poll, Frank van Rosmalen, Ruud Segers, Sander Steyns, Ulrich Strauch, Jeanette Tas, Iwan C. C. van der Horst, Sander M. J. van Kuijk, Dennis C. J. J. Bergmans

**Affiliations:** 1 Department of Intensive Care, Maastricht University Medical Centre+, P. Debyelaan 25, P.O. Box 5800, 6202 AZ Maastricht, The Netherlands; 2 Care and Public Health Research Institute (CAPHRI), Maastricht University, Maastricht, The Netherlands; 3 School of Nutrition and Translational Research in Metabolism (NUTRIM), Maastricht University, Maastricht, The Netherlands; 4 Department of Surgery, Maastricht University Medical Centre+, Maastricht, The Netherlands; 5 Department of Physiology, Maastricht University, Maastricht, The Netherlands; 6 Cardiovascular Research Institute Maastricht (CARIM), Maastricht University, Maastricht, The Netherlands; 7 Maastricht University Medical Centre+ Academy, Maastricht, The Netherlands; 8 Department of Respiratory Medicine, Maastricht University Medical Centre+, Maastricht, the Netherlands; 9 Center of Home Mechanical Ventilation, Maastricht University, Maastricht, The Netherlands; 10 School for Mental Health and Neuroscience (MHeNS), Maastricht University, Maastricht, The Netherlands; 11 Department of Clinical Epidemiology and Medical Technology Assessment, Maastricht University Medical Centre+, Maastricht, The Netherlands

**Keywords:** Respiratory distress syndrome, Outcomes research, Respiratory signs and symptoms

## Abstract

Patients with SARS-CoV-2 infection present with different lung compliance and progression of disease differs. Measures of lung mechanics in SARS-CoV-2 patients may unravel different pathophysiologic mechanisms during mechanical ventilation. The objective of this prospective observational study is to describe whether Electrical Impedance Tomography (EIT) guided positive end-expiratory pressure (PEEP) levels unravel changes in EIT-derived parameters over time and whether the changes differ between survivors and non-survivors. Serial EIT-measurements of alveolar overdistension, collapse, and compliance change in ventilated SARS-CoV-2 patients were analysed. In 80 out of 94 patients, we took 283 EIT measurements (93 from day 1–3 after intubation, 66 from day 4–6, and 124 from day 7 and beyond). Fifty-one patients (64%) survived the ICU. At admission mean PaO_2_/FiO_2_-ratio was 184.3 (SD 61.4) vs. 151.3 (SD 54.4) mmHg, (*p* = 0.017) and PEEP was 11.8 (SD 2.8) cmH_2_O vs. 11.3 (SD 3.4) cmH_2_O, (*p* = 0.475), for ICU survivors and non-survivors. At day 1–3, compliance was ~ 55 mL/cmH_2_O vs. ~ 45 mL/cmH_2_O in survivors vs. non-survivors. The intersection of overdistension and collapse curves appeared similar at a PEEP of ~ 12–13 cmH_2_O. At day 4–6 compliance changed to ~ 50 mL/cmH_2_O vs. ~ 38 mL/cmH_2_O. At day 7 and beyond, compliance was ~ 38 mL/cmH_2_O with the intersection at a PEEP of ~ 9 cmH_2_O vs. ~ 25 mL/cmH_2_O with overdistension intersecting at collapse curves at a PEEP of ~ 7 cmH_2_O. Surviving SARS-CoV-2 patients show more favourable EIT-derived parameters and a higher compliance compared to non-survivors over time. This knowledge is valuable for discovering the different groups.

## Introduction

Since the first report of the unusual cases of types of pneumonia attributed to Severe Acute Respiratory Syndrome Coronavirus-2 (SARS-CoV-2) in December 2019 in Wuhan, China; COVID-19, cases have surged across the world, causing considerable strain on the healthcare systems and unprecedented financial recession across the globe^1,2^. Mechanically ventilated SARS-CoV-2 patients admitted to the Intensive Care Unit (ICU) often require prolonged ventilator support and have a high risk of unfavourable outcome^3,4^. Studies have tried to unravel the underlying pathophysiology driving the lengthy need for ventilation support and investigate how to optimise ventilation to reduce duration and complications related to mechanical ventilation and favourable outcome^5,6^.

Since SARS-CoV-2 patients with similar oxygenation efficiency present with diverse lung compliance, pulmonary phenotypes have been proposed^7,8^. However, vigilance for premature phenotyping has been advocated since disease and pulmonary interaction might change the appearance of phenotype over time^9^. Nevertheless, heterogeneity of SARS-CoV-2 infection affecting the lung warrants tailored ventilation strategies. Most importantly, disease progression over time requires repeated titration of mechanical ventilation. Patient survival improves with higher positive end-expiratory pressure (PEEP) through reduction of atelectasis and recruitment of collapsed alveoli resulting in a decrease of driving pressure^10^, whereas excessive PEEP causes alveolar overdistension (OD) and ventilator-induced lung injury^11^, which is associated with lower patient survival^12^.

Electrical impedance tomography (EIT) is a clinical bedside technique to tailor mechanical ventilation by visualising aerated lung regions. EIT enables optimisation of PEEP by calculating both percentage OD and percentage alveolar collapse (CL). Optimisation of PEEP improves dynamic lung compliance (Cdyn) and may reduce the length of mechanical ventilation, complications, and mortality^13–16^. A case series of 15 mechanically ventilated patients with SARS-CoV-2 infection showed that a high PEEP level was required to reach low OD and low CL as determined by EIT^17^. However, whether a higher EIT-guided PEEP and a higher PaO_2_/FiO_2_-ratio over time are associated with survival remains unknown. Furthermore, case reports and case series of patients with SARS-CoV-2 infection have suggested that EIT can be used to indicate the presence of regional lung strain^18^, to decide on prone positioning or additional diagnostics in case of clinical deterioration^19–26^. These studies suggest that Cdyn, OD, and CL change over the course of mechanical ventilation during SARS-CoV-2 infection. How these pulmonary features behave over the course of mechanical ventilation remains uncertain.

We hypothesise that EIT-guided PEEP, OD, CL, and Cdyn change over the course of mechanical ventilation during SARS-CoV-2 infection. Moreover, EIT may identify heterogeneity of pulmonary pathophysiology that differs between survivors and non-survivors over time. Therefore, we conducted an observational study primarily focusing on serial measurements and report on EIT-derived pulmonary parameters over the course of mechanical ventilation in patients with SARS-CoV-2^27,28^.

## Methods

The patients’ characteristics, data collection, and the protocol for serial EIT measurements for this cohort have been described previously^27^.

### Participants

In the prospective MaastrICCht cohort, all consecutive COVID-19 patients at a tertiary teaching hospital in the Netherlands were included during the first pandemic wave (25.03.2020 until 12.06.2020). For the present study, we included invasive mechanically ventilated patients with a polymerase chain reaction positive for SARS-CoV-2 and a chest CT-scan scored positive based on a CORADS-score of 4–5 by a radiologist^29^. Patients were admitted via our emergency department, the non-ICU wards, and referral from other ICUs. Patients with a pacemaker or a body mass index ≥ 50 were excluded.

Our ICU has a capacity of 27 beds, divided over three subunits. To provide care for patients during the first wave of the COVID-19 pandemic, our ICU extended its capacity to a maximum of 64 beds. The medical ethical review board of the Maastricht UMC+ approved the study (review number 2020-1565/300523), which was performed in accordance with the declaration of Helsinki. During the pandemic, the board of directors of Maastricht UMC+ adopted a policy to inform patients or their legal representative and ask their consent to use their data for COVID-19 research purposes^27^. Informed consent was obtained for every patient. The study is registered in the Netherlands Trial Register (NL8613; 12/05/2020).

### Clinical protocol

All patients were ventilated in a pressure-controlled mode with tidal volumes around 6 ml/kg predicted body weight. No strict institutional guidelines on PEEP setting had been in use, and PEEP was set at the discretion of the treating physician based on clinical information and guidance according to a lung-protective ventilation strategy until the first EIT assessment.

### Daily clinical, physiological, and laboratory measurements

In addition to EIT measurements, arterial blood gas (ABG) analyses and ventilator settings before and after the PEEP trial were recorded in the daily clinical medical report form. Specifically, an ABG was collected before the PEEP trial (at 6 a.m. and if ventilator settings (besides FiO_2_) were changed between 6 a.m. and the EIT measurement) and half an hour after the PEEP trial. The ABG information combined with the FiO_2_ settings enable calculating the PaO_2_/FiO_2_-ratio to evaluate the effect of PEEP optimisation^30–33^.

### Serial chest electrical impedance tomography

An EIT belt was placed around the patients’ chest at the fourth or fifth intercostal space at the parasternal line and connected to an EIT monitor (Pulmovista® 500, Dräger Medical GmbH, Lübeck, Germany). Measurements were done in both prone and supine position. When a patient was turned from a supine to a prone position (or vice versa), the initiation of measurements was postponed at least 30 min to allow for stable mechanical ventilation conditions. To obtain accurate serial measurements, the position of the electrode belt was marked on the patients’ skin to ensure that each subsequent measurement was performed at the same position. The EIT monitor was connected to the ventilator to import all respiratory variables (e.g. tidal volume and Cdyn). The PEEP level was then increased until there was no further alveolar recruitment and solely OD, followed by a decremental PEEP trial, which was performed with steps of 2 cmH_2_O PEEP, preferably starting at 24 cmH_2_O. Each PEEP level was maintained for 30–60 s, and driving pressure was kept constant during the PEEP trial. However, the number of steps performed and the starting PEEP level could vary between patients. Hence, we present some data for PEEP levels beyond 24 cmH_2_O. Chest EIT measurements were recorded continuously during the decremental PEEP trial. The PEEP trial was discontinued if a significant CL was shown on the chest EIT images, a reduction of Cdyn, or a SpO_2_ < 88%. In addition to the chest EIT images, the percentage of CL and OD, tidal volume and Cdyn for each PEEP step were collected^27,34^. Importantly, chest EIT measurements were performed serially over the course of mechanical ventilation.

For the present study, any EIT data measured during Pressure support (PS) and Continuous positive airway pressure (CPAP) were excluded. The support ventilation modes increase the chance of informative missing data as serial EIT data on PS and CPAP are likely related to survival since these modes are indicative for weaning from mechanical ventilation. In addition, the EIT algorithm for calculating the level of OD and CL is not validated for PS and CPAP.

### Post-measurement

Optimal EIT-guided PEEP was determined at a level of CL ≤ 5% (supplementary Figure S1). If that level of PEEP simultaneously resulted in a high level of OD, driving pressure was decreased if possible. If a decrease in driving pressure was not possible, PEEP was set, allowing more CL closer to the intersection of OD and CL.

### Data analysis

#### Quality control and cleaning of chest EIT data

The EIT data quality is divided into good, intermediate, and poor quality (supplementary Table S1).

Supine vs. prone data: the maximum number of EIT measurements taken per day per patient was two (due to logistical reasons). This approach was applied if patients changed from supine to prone position or vice versa. For the present study, we report the main results of patients with good and intermediate quality EIT data in the supine position.

The data were processed to extract the data for each PEEP-step and prepared in MATLAB (Release 2019b, The MathWorks, Inc., Natick, Massachusetts, United States).

### Statistical analyses

All participants eligible for the study in the cohort during the first pandemic wave were included. The sample characteristics were described as mean and standard deviation (SD), median and interquartile range (IQR), or percentage, as appropriate. The statistical analysis was analysed with R version 3.6.1 using the R-studio interface.

Prior to the primary analyses, the course of PaO_2_/FiO_2_-ratios and PEEP levels over time was determined for those discharged from the ICU alive and those who died separately. Next, we used linear mixed-effects regression with a random intercept and random slope with time to compute differences in average PaO_2_/FiO_2_-ratio and PEEP level and differences in the slope over time between both groups. We used an unstructured variance–covariance matrix for the random effects and an autoregressive correlation structure of the first order for longitudinal measures. To assess non-linear change over time, we added polynomials of time. The best-fitting model for change over time was selected using the Akaike Information Criterion. The crude models were adjusted for age, sex, and APACHE II score.

For the primary analyses, after organising the serial EIT data into three levels, i.e., patient, time (days after intubation), and PEEP steps, we first used local polynomial regression fitting and cubic splines to construct population-based curves, with 95% confidence limits, for Cdyn mL/cmH_2_O, %CL, %OD for each of the PEEP steps from 24 to 8 cmH_2_O. We then categorised the serial EIT data into days after intubation: 1–3, 4–6, 7, and more, and used cubic splines to construct population-based curves for Cdyn, CL, and OD for these categories. Finally, with the categories days 1–3, days 4–6, and days 7 and more, and ICU survivors and ICU non-survivors, we used cubic splines to construct population-based curves for Cdyn, CL, and OD for these time categories for ICU survivors and ICU non-survivors. We chose this method to analyse the 3 level data, in contrast to the previously proposed mixed-models, as polynomial regression with cubic splines appeared to be a more flexible model for the 3 level data and used the previously proposed categories^27^.

We present means (solid line) with 95% CI (dashed lines) of %OD, %CL and Cdyn per PEEP step. *p*-value < 0.05 were considered statistically significant.

## Results

**Figure 1:**
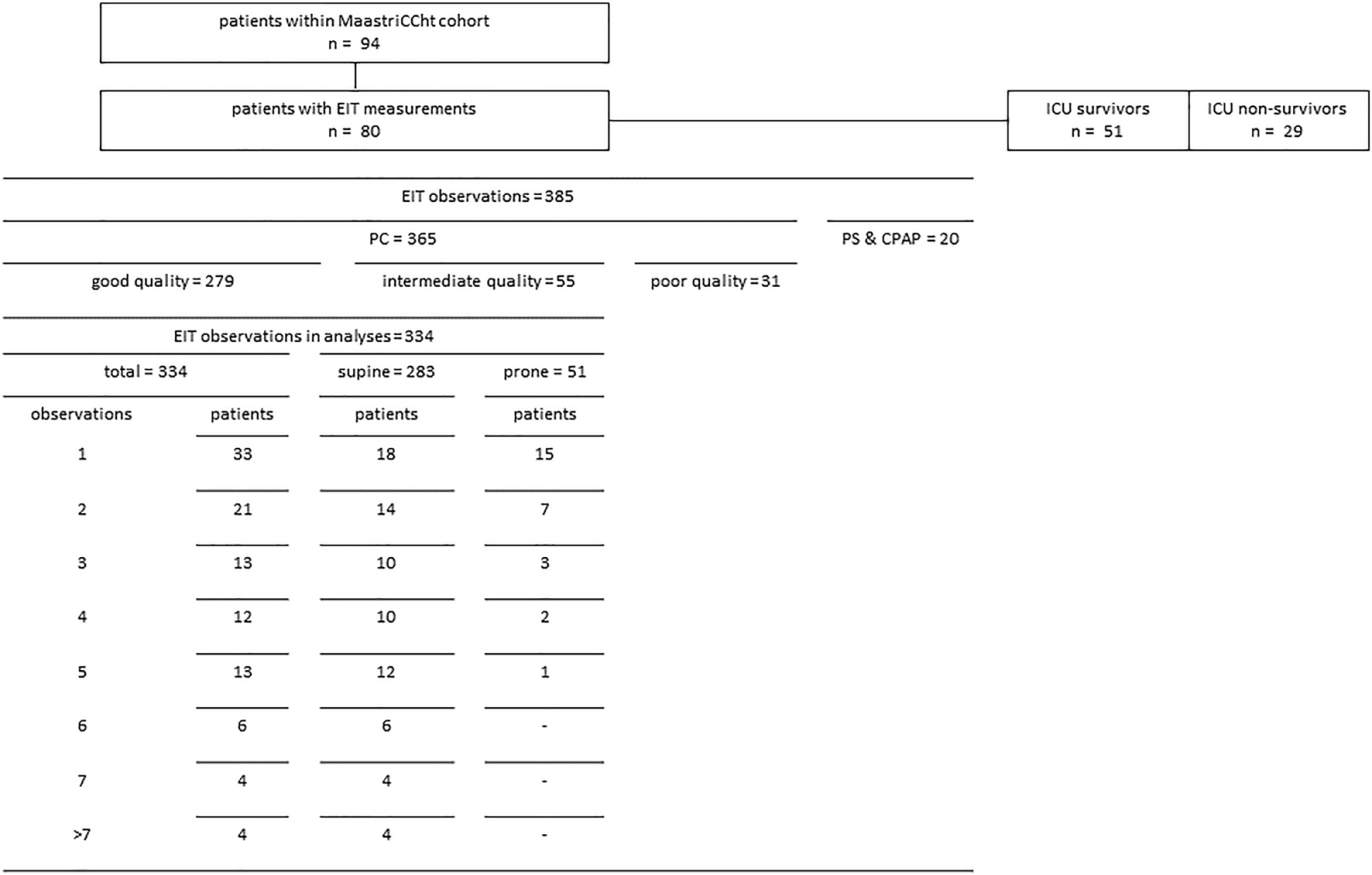
Study sample. Patients and good and intermediate quality serial EIT measurements during pressure control mode ventilation. Observations > 7 may include a range of 8 to 17 EIT observations, resulting in a total of 334. ICU, intensive care unit; EIT, electrical impedance tomography; PC, pressure control mode ventilation; PS, pressure support mode ventilation; CPAP, continuous positive airway pressure mode ventilation.

The *MaastrICCht* cohort comprises 94 patients included in the first pandemic wave (Fig. 1). Fourteen patients did not undergo EIT measurements due to a shorter length of stay (p < 0.001), see supplementary Table S2, while population characteristics were similar to those 80 patients who underwent EIT measurements. The mean age was 64.1 ± 11.6 years; 18 (22.5%) were female. The mean APACHE II score on admission was 16.1 ± 5.0 (Table 1). The mean duration of mechanical ventilation was 19.7 ± 13.0 days. After EIT data cleaning, 279 good quality and 55 intermediate quality EIT observations were included in the present study. Thirty-one poor observations were excluded (8.5% of total). Of all good and intermediate quality EIT observations, 283 (84.7%) were measured in the supine position and 51 (15.3%) in the prone position. Of the supine position EIT measurements, we included 93 observations from day 1 to 3 after intubation, 66 observations from day 4 to 6, and 124 observations beyond day 7. 51 (63.7%) participants survived the ICU. We experienced no significant haemodynamic side effects during the PEEP trials.

**Table 1:** Baseline demographic and clinical characteristics of the study population.

	**ICU survivors** **(*n* = 51)**	**ICU non-survivors** **(*n* = 29)**	***p*-value for difference**
Age, year	61.5 (11.9)	68.5 (9.9)	0.007
Sex, men	37 (72.5%)	25 (86.2%)	0.259
Body mass index, kg/m^2^	28.0 (4.4)	27.5 (4.0)	0.554
Chronic lung disease	2 (3.9%)	2 (6.9%)	0.618
APACHE II score, points	15.5 (5.1)	17.0 (4.7)	0.187
SOFA score, points	7.6 (2.6)	8.0 (2.2)	0.598
**Admission ventilation variables**			
Mechanical ventilation, n	48 (94.1%)	26 (89.7%)	0.662
Pressure control ventilation, n	34 (66.7)	23 (79.3)	0.307
FiO_2_, %	73.1 (18.8)	78.7 (18.0)	0.206
Respiration rate, per minute	22.7 (3.8)	22.1 (4.2)	0.680
Inspiratory pressure, cmH_2_O	27.0 (4.3)	27.5 (3.5)	0.754
PEEP, cmH_2_O	11.8 (2.8)	11.3 (3.4)	0.475
PaO_2_/FiO_2_-ratio	184.3 (61.4)	151.3 (54.4)	0.017
Tidal volume, ml/kg PBW	6.7 (1.1)	6.2 (1.2)	0.181
Arterial blood gas PaO_2_, kPa	10.5 (3.3)	10.4 (3.7)	0.876
Arterial blood gas PaO_2_, mmHg	79.0 (25.0)	78.0 (27.4)	0.876
Arterial blood gas PaCO_2_, kPa	5.9 (1.6)	6.3 (1.6)	0.399
Arterial blood gas PaCO_2_, mmHg	44.5 (12.1)	46.9 (12.1)	0.399
Arterial blood gas pH	7.36 (0.15)	7.28 (0.12)	0.025
Mean arterial blood pressure, mmHg	101.7 (12.7)	96.5 (15.6)	0.134
**Outcome variables**			
Length of mechanical ventilation, days	22.8 (13.8)	15.0 (9.9)	0.006

Data are means SD, median (IQR), and percentages as appropriate. ICU, Intensive Care Unit; APACHE II, Acute Physiology And Chronic Health Evaluation II; SOFA, serial organ failure assessment; FiO_2_, fraction of inspired oxygen; PEEP positive end-expiratory pressure; PBW, predicted body weight; PaO_2_/FiO_2_-ratio, oxygen arterial partial pressure on inspired fraction of oxygen ratio; PaO_2_, oxygen arterial partial pressure; PaCO_2_, carbon dioxide arterial partial pressure.

**Figure 2:**
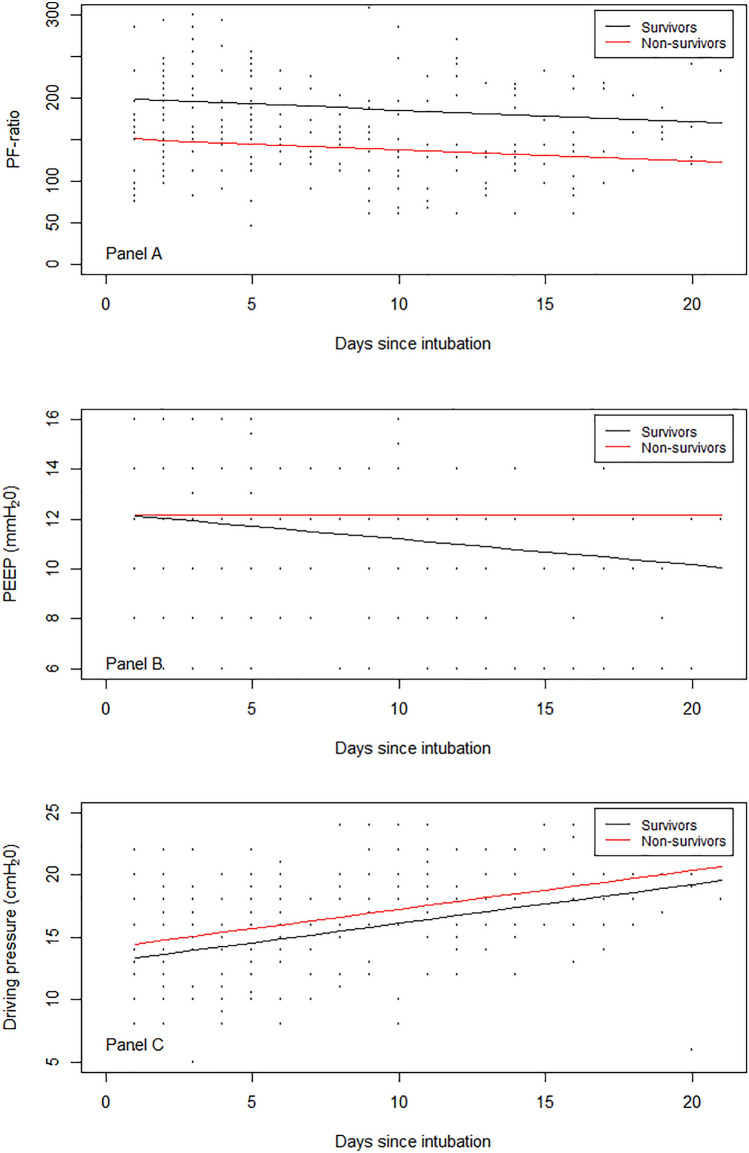
PaO_2_/FiO_2_-ratio (mmHg) and PEEP (cmH_2_O) for survivors and non-survivors over time. (**A**): PaO_2_/FiO_2_-ratio for survivors (black) and non-survivors (red) over time; (**B**): positive end-expiratory pressure (PEEP) for survivors and non-survivors over time; (**C**): driving pressure for survivors and non-survivors over time.

The mean PaO_2_/FiO_2_-ratio at baseline was 184.3 (SD 61.4) for ICU-survivors compared to 151.3 (SD 54.4) for those who died in the ICU (*p* = 0.017). The PEEP level at baseline was 11.8 (SD 2.8) cmH_2_O for ICU-survivors compared to 11.3 (SD 3.4) cmH_2_O for those who died in the ICU (*p* = 0.475). Figure 2 shows the observed PaO_2_/FiO_2_-ratio (panel A), PEEP level (panel B), and driving pressure (panel C) for ICU-survivors and ICU-non-survivors throughout follow-up with lines superimposed showing the best-fitting overall trajectories over time, unadjusted for confounders. On average, ICU-survivors had a higher overall PaO_2_/FiO_2_-ratio during their ICU stay (regression coefficient: 47.4, 95% CI: 26.6; 68.3, *p* < 0.001) after adjustment for age, sex, and APACHE II score. A model including an interaction-term between mortality and time revealed no significant interaction by time (*p* = 0.471). On average, ICU-survivors had a non-statistically significant lower overall mean PEEP during their ICU stay (regression coefficient: − 0.53, 95% CI: − 1.98; 0.92, *p* = 0.473) after adjustment for age, sex, and APACHE II score. A model including an interaction-term between mortality and time revealed a significant interaction by time (*p* = 0.026), meaning a steeper decrease for survivors compared to non-survivors over time. There was no evidence of an average difference in overall mean driving pressure between ICU-survivors and non-survivors during their ICU stay (regression coefficient: − 1.37, 95% CI: − 2.98; 0.23, *p* = 0.096) after adjustment for age, sex, and APACHE II score.

### EIT population curves

**Figure 3:**
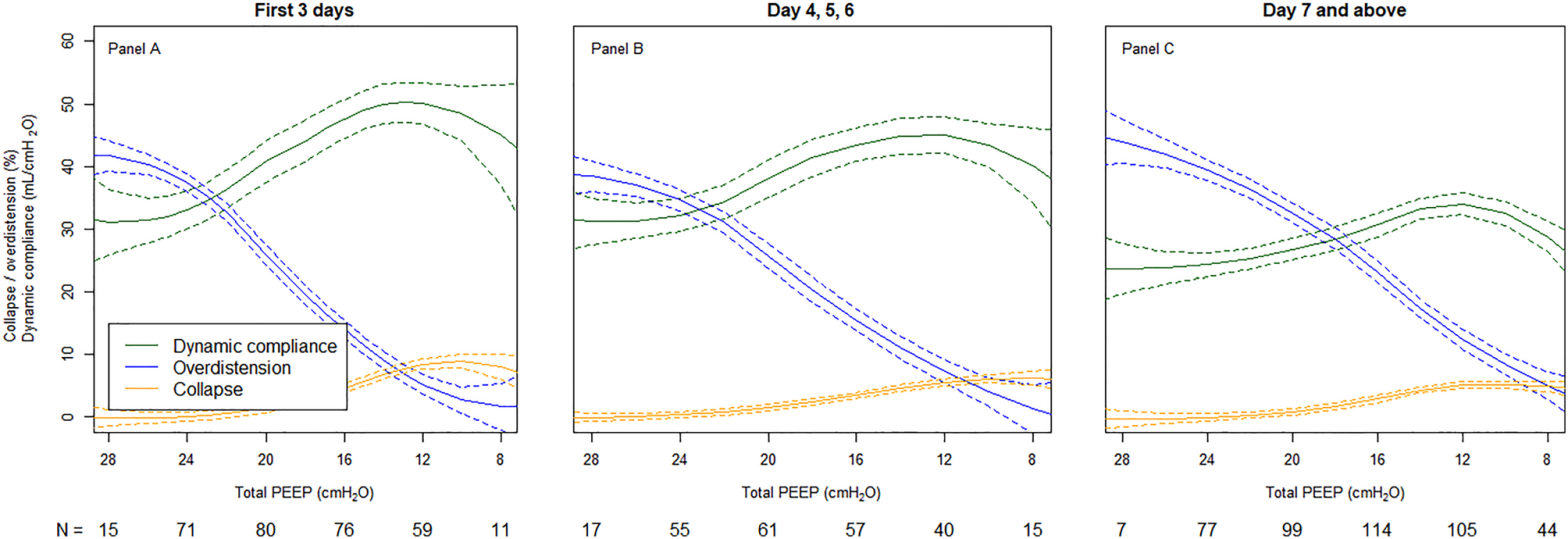
Dynamic respiratory system compliance, alveolar overdistension, and alveolar collapse for the whole population from admission to beyond a week after. EIT population curves that show mean (solid lines), dynamic compliance (green), overdistension (blue), and collapse (yellow) with 95% confidence intervals (dashed lines) for the whole population from PEEP steps 28 to 8 cmH_2_O over time for the first 3 days (**A**), day 4, 5 and 6 (**B**) and day 7 and above (**C**).

Figure 3 shows OD decreased with a lesser slope over time, and CL decreased from ~ 10 to ~ 7% over time (panel A: day 1–3; panel B: day 4–6; panel C: day ≥ 7, respectively). The combination of these changes shifted the intersection between OD and CL curves to the right (lower PEEP) over time. Maximal Cdyn decreased from ~ 50 mL/cmH_2_O (panel A: day 1–3) to ~ 35 mL/cmH_2_O (panel C: day ≥ 7) over time.

**Figure 4:**
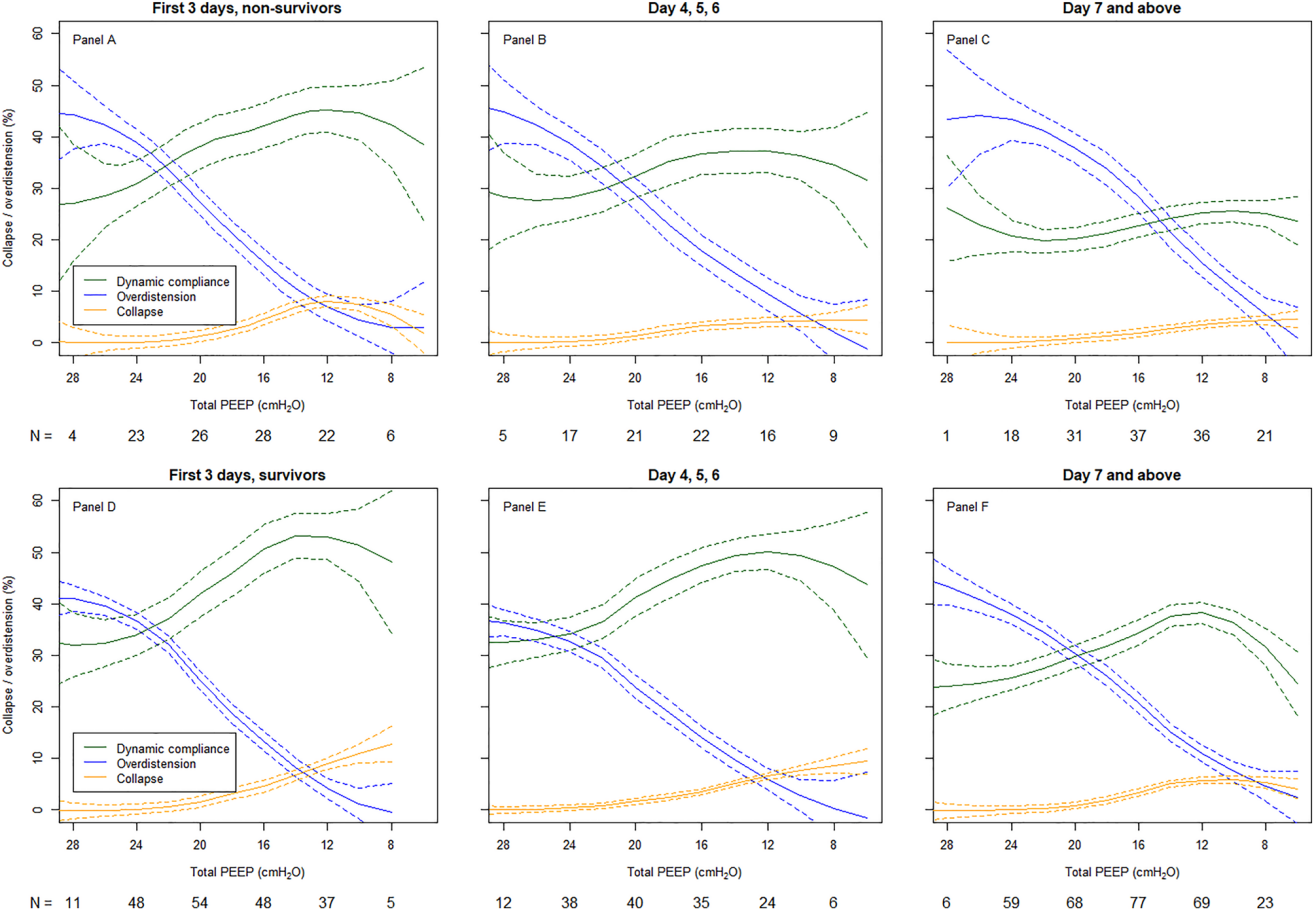
Dynamic respiratory system compliance, alveolar overdistension, and alveolar collapse for the whole population from admission to beyond a week after in ICU survivors and ICU non-survivors. EIT population curves that show mean (solid lines), dynamic compliance (green), overdistension (blue), and collapse (yellow) with 95% confidence intervals (dashed lines) from PEEP steps 28 to 8 cmH_2_O for ICU non-survivors over time for the first 3 days (**A**), for day 4, 5 and 6 (**B**) and for day 7 and above (**C**); and for ICU survivors over time for the first 3 days (**D**), for day 4, 5 and 6 (**E**) and for day 7 and above (**F**).

Figure 4, panels A and D for days 1–3, show ICU non-survivors (upper panel) had a maximal Cdyn of ~ 45 mL/cmH_2_O, and ICU survivors (lower panel) had a Cdyn of ~ 55 mL/cmH_2_O, while the intersection of OD and CL curves appeared similar at a PEEP of ~ 12–13 cmH_2_O. Figure 4, panels B and E show ICU non-survivors had a maximal Cdyn of ~ 38 mL/cmH_2_O, and ICU survivors had a Cdyn of ~ 50 mL/cmH_2_O, while the intersection between OD and CL curves shifted to the right for ICU non-survivors to a PEEP of ~ 10 cmH_2_O and remained at a PEEP of ~ 12–13 cmH_2_0 for ICU survivors for day 4–6. Figure 4, panels C and F show ICU non-survivors had a maximal Cdyn of ~ 25 mL/cmH_2_O with an intersection between OD and CL curves at a PEEP of ~ 7 cmH_2_O, and ICU survivors had a Cdyn of ~ 38 mL/cmH_2_O with an intersection between OD and CL curves at a PEEP of ~ 9 cmH_2_O for day 7 and thereafter.

### Additional analyses

We show that in the 283 EIT measurements, OD decreased from ~ 40 to ~ 5%, CL increased from 0 to ~ 8%, with a maximal Cdyn of ~ 40 mL/cmH_2_O at a PEEP of ~ 13 cmH_2_O during PEEP steps from 28 to 8 cmH_2_O (supplementary Figure S2).

Repeating the analyses for PEEP steps above 28 cmH_2_O showed increasingly less confidence (widening of dashed lines with higher PEEP) than for PEEP < 24 cmH_2_O (supplementary Figure S3). Repeating the analyses, including EIT data for prone positioning, only showed that OD, CL, and Cdyn curves, on average, including all time points and all PEEP steps, differ from OD, CL, and Cdyn in the supine position (supplementary Figure S4). Stratifying results for men and women showed that the low amount of data for women, on average, led to less precise estimates of OD, CL, and Cdyn curves (supplementary Figure S5).

## Discussion

The present study in SARS-CoV-2 patients on mechanical ventilation has four main findings. First, we show that using EIT guided PEEP; OD, CL, and Cdyn change over the course of mechanical ventilation during SARS-CoV-2 infection in a way that suggests that the whole population develops decreased compliance over time. Second, we show that these changes are more unfavourable in non-survivors than survivors. More precisely, over the course of mechanical ventilation, the intersection between OD and CL shifted towards the right which, together with the decreasing Cdyn indicates stiffening of the lungO. Third, after adjustment for age, sex, and APACHE II score, a higher PaO_2_/FiO_2_-ratio over time, but not PEEP or driving pressure, was associated with survival. Overall, this suggests that PEEP titration seems to be important both on an individual basis and over time. Fourth, our results show that the unfavourable pattern (i.e., lower Cdyn with higher OD and CL) over time is observed mainly in non-survivors, suggesting that stiffening of the lung over time by COVID-19 related pro-fibrotic processes^35^ and/or ventilator-induced injury, is associated with unfavourable outcome.

Our prospective cohort study with serial EIT measurements in mechanically ventilated patients with SARS-CoV-2 infection is comprehensive and adds to the previous reports^17,19–24,33,36–42^. Additional to previous studies, we included much more patients with SARS-CoV-2 infection^17,19–24,42–45^ or acute respiratory distress syndrome^33,36–40^, most innovative of this study is that we performed a unique amount of serial EIT measurements (334 EIT observations)^17,24,33,36–40,42,43^ and mainly included patients with more than 1 PEEP trial per patient (supplementary Table S3)^19–22,24^. Moreover, OD, CL, and Cdyn were analysed on the population level using serial measurements to provide more in-depth information on lung pathophysiology over time.

In our centre, optimised EIT-guided PEEP is titrated to a CL ≤ 5%. Other centres titrate PEEP at the intersection of OD and CL. Momentarily, there is no gold standard. However, the best Cdyn in our population is always in the range between CL ≤ 5% and the intersection of OD and CL (supplementary Figure S2).

Our results are similar to van der Zee et al., who showed that high optimum PEEP was required to reach a low OD and low CL by EIT^17^ and we extend the scarce serial data^19–22^. We add that the interrelationship between optimum PEEP, OD, CL, and Cdyn changed over time, showing worse lung dynamics in non-survivors beyond 7 days of mechanical ventilation compared to 1–3 and 4–6 days, respectively. The flattening of the Cdyn curve during the PEEP trial in the non-survivors beyond day 7 indicates the progression of the amount of non-recruitable lung tissue. Furthermore, reducing PEEP in this population does not increase CL. Our results provide evidence for a pathophysiological framework that stiffens the lung during mechanical ventilation over time in a way that leads to an unfavourable outcome. Profibrotic processes, due to increased TGF-β in COVID-19 among other mechanisms^35^, thus likely play a role in affecting outcome and persistent post-discharge sequelae^46^. Whether intervention is possible remains unclear. Another explanation might be that lung stiffening results from ventilator-induced injury which in this study is minimized by EIT PEEP optimisation but cannot be totally avoided^47^. We did not investigated recruitability in this paper. But in our experience, only a small number of patients were responders to recruitment in the early phase after intubation. This effect decreased over time. In general, most patient had a high compliance compared to their PaO_2_/FiO_2_-ratio at the start of mechanical ventilation. This may explain the low recruitability. Later in the progress of the disease, compliance decreased and stiffening of the lung occurred. Resulting further in a decreased recruitability which is also illustrated by our finding that reducing PEEP in this population does not increase CL.

For the main analyses, we reported on EIT data determined in patients on mechanical ventilation using PC in a supine position. Additionally, we analysed EIT parameters stratified for prone positioning separately, which is important when investigating Cdyn, OD, and CL over time as prone and supine positioning appeared to affect these parameters differently. Similarly, we additionally stratified the results for men and women. Nevertheless, the amount of data on Cdyn, OD, and CL for women and prone positioning was too small to conclude on in these subgroups based on a population level and over time, respectively.

The strengths of the study include its prospective design and the predefined protocol for serial EIT measurements. Furthermore, to study the course of OD, CL, and Cdyn over time, we used the date of intubation as a starting point. This minimises information bias due to the timing of EIT measurements. The large amount of data increases the precision of the population EIT curves, illustrated by the narrow 95% confidence limits.

Limitations of the study are the inclusion of SARS-CoV-2 infected patients only, which may limit the generalisability of the results to other pulmonary pathology^33,36–40^. Furthermore, we cannot fully exclude that selection has biased the results as patients were transported between hospitals during the pandemic. However, both the most severe critically ill patients were transported to our institution, for example, when advanced pulmonary support by extracorporeal membrane oxygenation techniques was considered, and the less critically ill patients, because transport was considered most feasible in those patients. Most likely, this averages the severity of pulmonary pathology and critical illness within our population. Patients transported from other centres are likely to be intubated for some time, and thus they may differ in the disease course. Nevertheless, the serial EIT data were aligned from intubation to match the disease courses between patients. Furthermore, although sex-differences likely play a role in COVID-19 pathophysiology^28,48^, the stratified results for women should be interpreted with caution due to lack of precision. In fact, this result illustrates the importance of a major amount of data when investigating EIT curves on a population level. Finally, we have described observational results, which limit conclusions with regards to causality.

In conclusion, we show that EIT guided PEEP, OD, CL, and Cdyn change over the course of mechanical ventilation during SARS-CoV-2 infection in a way that suggests that all patients develop decreased compliance over time. These changes are more unfavourable in non-survivors as compared to survivors. This suggests that PEEP titration might be valuable not only on an individual basis but also over time; the exact clinical relevance remains to be established. Moreover, we found that a higher PaO_2_/FiO_2_-ratio over time, but not PEEP or driving pressure, was associated with survival. Furthermore, these results provide a pathophysiological framework for mechanisms that stiffen the lung contributing to a more unfavourable pattern (i.e., lower Cdyn with higher OD and CL) over time in relation to adverse outcome in COVID-19 patients on mechanical ventilation.

## Supplementary Information

Supplementary Information.

## Data Availability

The datasets generated during and/or analyzed during the current study are available from the corresponding author on reasonable request.

## Abbreviations

Cdyn: Dynamic respiratory system compliance
CL: Alveolar collapse
CPAP: Continuous positive airway pressure
EIT: Electrical impedance tomography
ICU: Intensive Care Unit
OD: Alveolar overdistension
PC: Pressure control
PEEP: Positive end-expiratory pressure
PS: Pressure support
