# Supplementary material for: Pulmonary pathophysiology development of COVID-19 assessed by serial Electrical Impedance Tomography in the MaastrICCht cohort
Source: Sci Rep. 2022 Aug 25;12:14517. doi: 10.1038/s41598-022-18843-z (PMC9403977; doi:10.1038/s41598-022-18843-z)
Supplement: Supplementary file 1 — Supplementary Information. [file 41598_2022_18843_MOESM1_ESM.docx]

**Pulmonary pathophysiology development of COVID-19 assessed by serial Electrical Impedance Tomography in the MaastrICCht cohort**

**Supplementary Material**

*Journal*

Scientific Reports

*Authors*

Serge J.H. Heines, Bas C.T. van Bussel, Melanie J. Acampo-de Jong, Frank C. Bennis, Rob J.J. van Gassel, Rald V.M. Groven, Nanon F.L. Heijnen, Ben J.M. Hermans, René Hounjet, Johan van Koll, Mark M.G. Mulder, Marcel C.G. van de Poll, Frank van Rosmalen, Ruud Segers, Sander Steyns, Ulrich Strauch, Jeanette Tas, Iwan C.C. van der Horst, Sander M.J. van Kuijk, Dennis C.J.J. Bergmans

*Corresponding author*

S. Heines, Department of Intensive Care, Maastricht University Medical Centre+, Maastricht, The Netherlands

P. Debyelaan 25, 6202 AZ Maastricht, The Netherlands, E-mail: s.heines@mumc.nl

**Content**

Supplemental figures: 5

Supplemental tables: 3

**Supplementary** **Figure 1:** Titration of positive end expiratory pressure (PEEP) with cumulated regional collapse and overdistension based on EIT.

The top row shows the decremental PEEP steps, whereas the second row shows ventilation distribution from blue to white (the whiter the more ventilation distribution). The third row shows relative alveolar overdistension (orange) and relative alveolar collapse (white). The percentages of relative overdistension and collapse are presented in the lowest graph. RVD = regional ventilation delay, CL HP (%) = compliance loss high pressure, CL LP (%) = compliance loss low pressure.

**
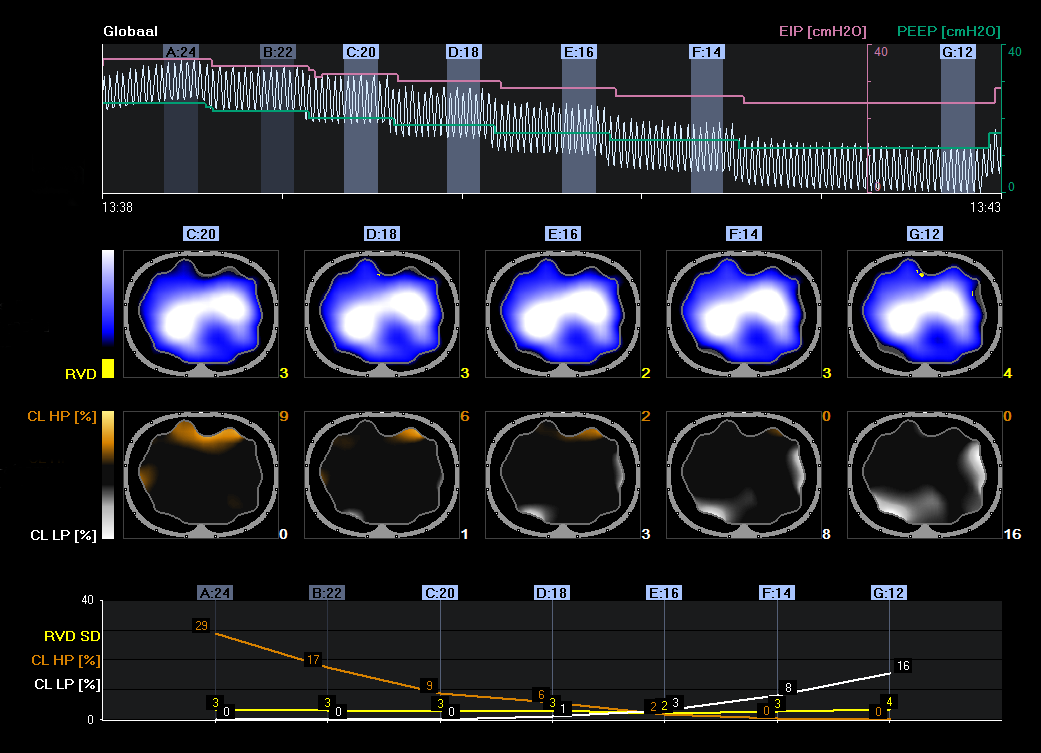
**

**Supplementary** **Figure 2:** Dynamic respiratory system compliance, alveolar overdistension, and alveolar collapse for the whole population during the decremental PEEP trial.

EIT population curves show mean (solid lines), dynamic compliance (green), overdistension (blue), and collapse (yellow) with 95% confidence intervals (dashed lines) for the whole population and all time points from PEEP steps 28 to 8 cmH_2_0.


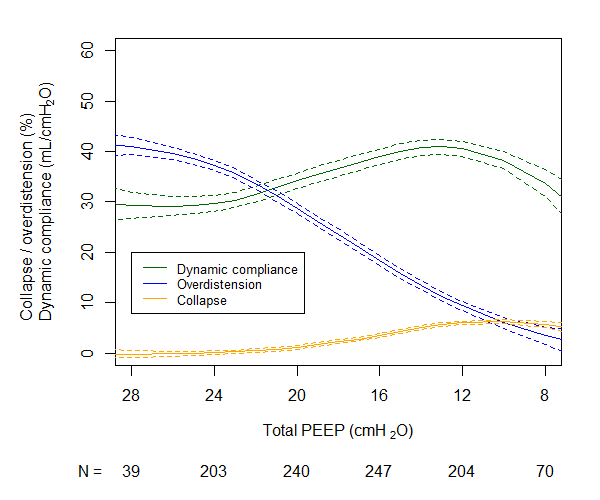


**Supplementary** **Figure 3:** EIT population curves for the whole population.

EIT population curves that show mean (solid lines), dynamic compliance (green), overdistension (blue), and collapse (yellow) with 95% confidence intervals (dashed lines) for the whole population and all time points from PEEP steps 36 to 4 cmH_2_0.


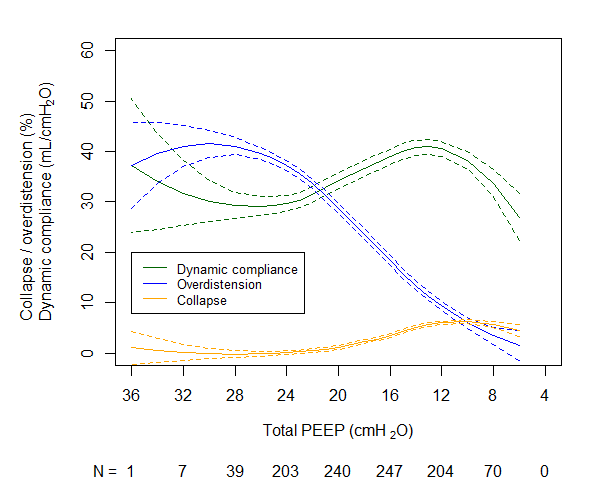


**Supplementary** **Figure 4:** EIT population curves for prone position.

EIT population curves show mean (solid lines), dynamic compliance (green), overdistension (blue), and collapse (yellow) with 95% confidence intervals (dashed lines) for prone position only, including all time points from PEEP steps 36 to 4 cmH_2_0 combined. The results include 51 serial measurements in PC mode ventilation in the prone position.


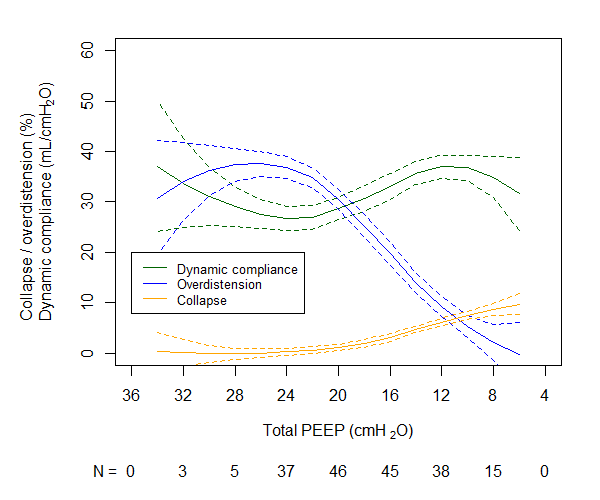


**Supplementary** **Figure 5**: EIT population curves stratified for men and women.

EIT population curves show mean (solid lines), dynamic compliance (green), overdistension (blue), and collapse (yellow) with 95% confidence intervals (dashed lines) stratified for men (panel A) and women (panel B), including all time points from PEEP steps 28 to 8 cmH_2_0.


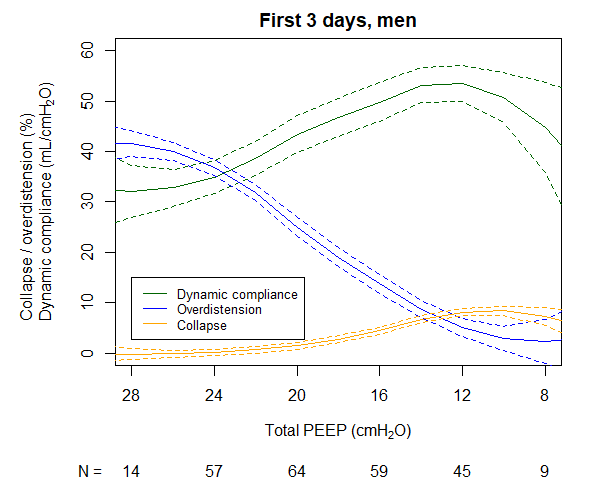


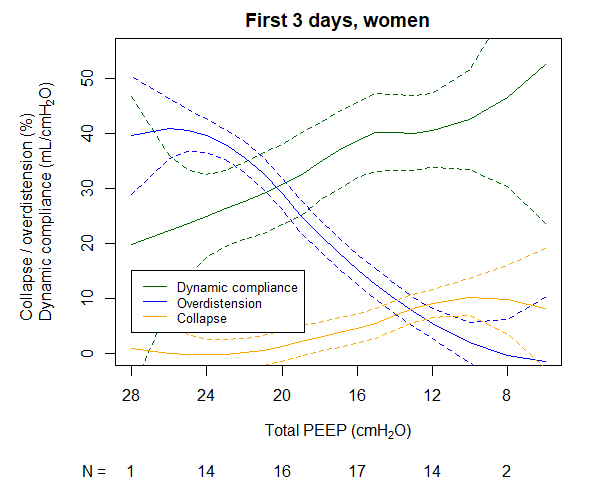


**Supplementary** **Table 1: Quality control and cleaning of chest EIT data.**

Measurements were classified as good, intermediate, or poor quality by detecting alveolar overdistension and alveolar collapse errors between steps of the decremental PEEP trial. Lung physiology defines that a reduction in PEEP for each decremental PEEP step causes a physiological decrease of overdistension and a physiological increase in collapse^E1^.

| **Good quality** | A linear progression of alveolar overdistension (decrease) and alveolar collapse (increase) during the decremental PEEP trial was observed. |
| --- | --- |
| **Intermediate quality** | Compared to the previous decremental PEEP step change, a decreased alveolar overdistension or an increased alveolar collapse of less than 15% (arbitrarily defined by the researchers) was observed. This means that the changes in alveolar overdistension and alveolar collapse evoked by a decremental PEEP step were physiologically in the right direction but relatively large compared to the previous changes between PEEP steps. This resulted in a slight deviation from linear progression of overdistension and collapse during the decremental PEEP trial. |
| **Poor quality** | An increased alveolar overdistension or a decreased alveolar collapse was observed. This is physiologically impossible while lowering PEEP over the decremental PEEP trial. In addition, a decreased overdistension or an increased collapse of more than 15% compared to the previous decremental PEEP step also defined poor EIT measurement quality, meaning that the changes in overdistension and collapse evoked by a decremental PEEP step were physiologically in the right direction but too large compared to the previous changes between PEEP steps, resulting in a large deviation from linear progression of overdistension and collapse during the decremental PEEP trial. |

^E1^Gomez-Laberge C, Arnold JH, Wolf GK. A unified approach for EIT imaging of regional overdistension and atelectasis in acute lung injury. IEEE Trans Med Imaging. 2012;31(3):834-42

**Supplementary** **Table 2: Baseline demographic and clinical characteristics of the cohort divided into those selected for this study and those without EIT measurements performed.**

| **Mean (SD) or Median (IQR)** | **EIT performed**  **(*n* = 80)** | **No EIT performed**  **(*n* = 14)** | ***p*-value** |
| --- | --- | --- | --- |
| Age, year | 64.1 (11.6) | 65.9 (13.3) | 0.625 |
| Sex, men | 62 (77.5%) | 12 (85.7%) | 0.735 |
| Body mass index, kg/m^2^ | 27.8(4.3) | 26.4 (2.6) | 0.119 |
| Chronic lung disease | 4 (5.0%) | 4 (28.6%) | 0.016 |
| APACHE II score, points | 16.0 (5.0) | 14.6 (9.0) | 0.567 |
| SOFA score, points | 7.7 (2.5) | 8.7 (3.5) | 0.409 |
| **Admission ventilation variables** |  |  |  |
| Mechanical ventilation, *n* | 74 (92.5%) | 13 (92.9%) | 1.000 |
| Pressure control ventilation, *n* | 24 (25.5%) | 7 (50.0%) | 0.216 |
| FiO_2_, % | 75.1 (18.6) | 66.2 (18.6) | 0.129 |
| Respiration rate, per minute | 22.5 (3.9) | 18.3 (8.6) | 0.248 |
| Inspiratory pressure, cmH_2_O | 27.2 (3.9) | 25.0 (4.4) | 0.343 |
| PEEP, cmH2O | 11.7 (3.4) | n.a. | n.a. |
| PaO_2_/FiO_2_-ratio | 110.7 (48.1) | 139.2 (67.3) | 0.166 |
| Tidal volume, ml/kg PBW | 6.5 (1.1) | 6.7 (1.5) | 0.818 |
| Arterial blood gas PaO_2_, kPa | 10.5 (3.4) | 11.7 (5.4) | 0.424 |
| Arterial blood gas PaO_2_, mmHg | 78.4 (25.7) | 88.0 (40.5) | 0.424 |
| Arterial blood gas PaCO_2_, kPa | 6.0 (1.6) | 5.7 (2.3) | 0.675 |
| Arterial blood gas PaCO_2_, mmHg | 45.1 (12.2) | 43.0 (17.4) | 0.675 |
| Arterial blood gas, pH | 7.3 (0.1) | 7.3 (0.2) | 0.600 |
| Mean arterial blood pressure, mmHg | 99.8 (13.9) | 89.6 (11.7) | 0.009 |
| Length of mechanical ventilation, days | 19.7 (13.0) | 12.3 (17.0) | 0.238 |

Data are expressed as means SD, median (IQR) and percentages as appropriate. APACHE II, Acute Physiology And Chronic Health Evaluation II; SOFA, serial organ failure assessment; FiO_2_, fraction of inspired oxygen; PEEP, positive end-expiratory pressure; PaO_2_/FiO_2_-ratio, arterial partial pressure of O_2_ and fraction of inspired oxygen ratio; PBW, predicted body weight; PaO_2_, partial pressure of oxygen; PaCO_2_, partial pressure of carbon dioxide; ICU, Intensive Care Unit; N.a., not any number; SD, standard deviation; IQR, interquartile range.

**Supplementary** **Table 3**: Studies on EIT in patients with COVID-19.

| **Reference** | **Design** | **COVID-19, n** | **Non-COVID-19, n** | **EIT Technique** | **Clinical method** | **Primary outcome** | **Best PEEP COVID-19** | **Best PEEP Non-COVID-19** | **Additional information** |
| --- | --- | --- | --- | --- | --- | --- | --- | --- | --- |
| Perier F, Crit Care 2020 [42] | Cohort | 17 | 13 | Enlight 1800 (Timpel SA) | Smallest sum of hyperdistension and collapse | N.A. | 12 [9,12] median [IQR] | 9 [6,9] median [IQR] | Best PEEP similar in supine and prone |
| Van der Zee P, AJRCCM 2021 [15] | Case series | 15 | - | Pulmovista 500 (Dräger Med.) or Enlight 1800 (Timpel SA) | PEEP was set at the PEEP level above the intersection of the overdistension and collapse curves | EIT vs. PEEP-FiO_2_ table | 21 [16,22] median [IQR] |  | EIT guided PEEP was higher compared to the low and higher PEEP-FiO_2_ table |
| Mauri T, CCM 2020 [40] | Crossover physiologic study | 10 | - | Pulmovista 500 (Dräger Med.) | V/Q matching and ventilation inhomogeneity in different PEEP levels | N.A. |  |  | Potential for lung recruitment presents large variability, while elevated dead space may guide mechanical ventilation |
| Perier F, AJRCCM 2020 [43] | Ancillary report of 2 ongoing studies | 9 | - | Enlight 1800 (Timpel SA) | The effect of PEEP and prone position on V/Q matching | N.A. |  |  | Prone position and increased PEEP shifted ventilation but did not change perfusion |
| Sella N, Crit Care 2020 [19] | Case series | 5 | - | Pulmovista 500 (Dräger Med.) | Best compromise between lung collapse and overdistension | EIT vs. PEEP table | 12 [10,14] median [IQR] |  | Compared to the higher and lower PEEP-FiO_2_ table, EIT PEEP was lower and higher, respectively |
| MIcek M, BMCPM 2021 [21] | Case series | 5 | - | Enlight 1800 (Timpel SA) | The effect of optimal PEEP titration in supine vs. targeted lateral position on lung collapse and overdistension | N.A. |  |  | Targeted lateral positioning with bedside personalised PEEP provided a selective attenuation of  overdistension and collapse |
| Morais C, AJRCCM 2020 [41] | Case series | 3 | - | Enlight 1800 (Timpel SA) | V/Q matching and ventilation distribution | N.A. |  |  | One case showed severe V/Q mismatch with normal ventilation distribution and high compliance, pulmonary embolism was confirmed |
| Taenaka H, RMCR 2021 [22] | Case report | 2 | - | BB^2^ (Swisstom AG) | The effect of supine vs. prone position and low vs. high PEEP on oxygenation and alveolar collapse | N.A. |  |  | Prone position improved oxygenation and reduced alveolar collapse, visualisation of silent spaces by EIT was useful to predict optimal PEEP |
| Tomasino S, SCVA 2020 [17] | Case report | 2 | - | Pulmovista 500 (Dräger Med.) | Best ventilation distribution and homogeneity after prone position | N.A. |  |  |  |
| Zarantonello F, AJRCCM 2020 [20] | Case report | 1 | - | Pulmovista 500 (Dräger Med.) | The effect of prone position on V/Q matching | N.A. |  |  | Ventilation increased after 1 hour of pronation, perfusion decreased |
| Zhao Z BMC Pulm Med 2021 [39] | Case report | 1 | - | N.A. | Cumulated overdistension and collapse, ventilation distribution at two-time points, changes in end expiratory lung impedance | N.A. |  |  | Patients’ response to PEEP titration, body position, ventilator support level |
| Fakhr B, BJA 2020 [18] | Case report | 1 | - | Enlight 1800 (Timpel SA) | V/Q matching to confirm pulmonary thrombosis | N.A. |  |  | Imbalance in lung perfusion despite even distribution of ventilation |
| Pulletz, J Clin Monit Comput 2021 [16] | Observational study | 10 | 10 | BB^2^ (Swisstom AG) | Dynamic relative regional strain was defined as the ratio of tidal impedance changes and end-expiratory lung impedance within each pixel of the lung region to indicate the presence of P-SILI | N.A. |  |  | Patients with oxygen therapy via nasal cannula or high flow nasal oxygen therapy versus healthy volunteers |
| Fu, Physiol Meas 2020 [24] | Case report | 1 |  | Pulmovista 500 (Dräger Med.) | Regional ventilation changes before BAL, 30 min after and in the following days, were monitored with EIT | N.A. |  |  | Therapeutic BAL might improve regional ventilation for COVID-19 |
| Shono, J Crit Care Med 2021 [23] | Case report | 1 |  | Pulmovista 500 (Dräger Med.) | Combination of increase in end-expiratory lung impedance and ventral distribution of ventilation in the dorsal lung region | N.A. | day 2 (14), day 3 (15), day 5 (15) |  | Patient had 4 prone positioning sessions, EIT was only performed in supine position |

EIT, electrical impedance tomography; PEEP, positive end expiratory pressure; FiO_2_, inspired fraction of oxygen; IQR, interquartile range; V/Q matching, ventilation/perfusion ratio; N.A., not available
